# Nonphotodynamic Roles of Methylene Blue: Display of Distinct Antimycobacterial and Anticandidal Mode of Actions

**DOI:** 10.1155/2018/3759704

**Published:** 2018-01-31

**Authors:** Rahul Pal, Moiz A. Ansari, Venkata Saibabu, Shrayanee Das, Zeeshan Fatima, Saif Hameed

**Affiliations:** Amity Institute of Biotechnology, Amity University Haryana, Manesar, Gurugram 122413, India

## Abstract

Significance of methylene blue (MB) in photodynamic therapy against microbes is well established. Previously, we have reported the antifungal potential of MB against* Candida albicans*. The present study attempts to identify additional antimicrobial effect of MB against another prevalent human pathogen,* Mycobacterium tuberculosis* (MTB). We explored that MB is efficiently inhibiting the growth of* Mycobacterium* at 15.62 *μ*g/ml albeit in bacteriostatic manner similar to its fungistatic nature. We uncovered additional cell surface phenotypes (colony morphology and cell sedimentation rate) which were impaired only in* Mycobacterium*. Mechanistic insights revealed that MB causes energy dependent membrane perturbation in both* C. albicans* and* Mycobacterium*. We also confirmed that MB leads to enhanced reactive oxygen species generation in both organisms that could be reversed upon antioxidant supplementation; however, DNA damage could only be observed in* Mycobacterium*. We provided evidence that although biofilm formation was disrupted in both organisms, cell adherence to human epithelial cells was inhibited only in* Mycobacterium*. Lastly, RT-PCR results showed good correlation with the biochemical assay. Together, apart from the well-established role of MB in photodynamic therapy, this study provides insights into the distinct antimicrobial mode of actions in two significant human pathogens,* Candida* and* Mycobacterium,* which can be extrapolated to improve our understanding of finding novel therapeutic options.

## 1. Introduction

In this era of antibiotics, many efficient drugs have been developed or are under development to treat various infectious diseases. But the real challenge is to surmount the parallel evolution of drug resistance, which is also emerging at similar pace [1, 2]. Cumulatively, both bacterial and fungal infections are responsible for the major cause of mortality in the world. Among bacterial infections, tuberculosis (TB) is a frequent infectious disease in human, caused by* Mycobacterium tuberculosis* (MTB) and a brutal killer bacterium responsible for millions of deaths annually around the globe [2]. Similarly, among fungal infections, due to its opportunistic nature,* Candida albicans* is one of the major fungal pathogens that has emerged in the immunocompromised conditions such as AIDS, diabetes, organ transplantation, burn [3, 4], and coinfection with organisms such as MTB [2]. The length and complexity of currently available chemotherapy for TB and fungal infections are far from satisfactory due to high costs, toxicity, and emergence of multidrug resistance (MDR) strains of MTB and* C. albicans* which is causing a menace to the current therapeutic regime. Hence, the need for the alternative therapeutics with efficiency to restrain the growth of both microbes is indispensable.

Methylene blue (MB) is widely used as dye in variety of biological sciences applications [5, 6]. MB has been used for diagnostic procedures and the treatment of multiple disorders, including methemoglobinemia, cyanide, and carbon monoxide poisoning, and is considered to be nontoxic [7]. Moreover, due to its light absorbing nature, MB has been extensively used in photodynamic therapy [8]. Previously we have demonstrated the antifungal potential of MB against* C. albicans* [9]. This study uncovers additional targets of MB in* C. albicans* and not only reveals deeper insights into the anticandidal mechanisms but also provided sufficient clues to decipher its antimycobacterial potential against the surrogate model of MTB,* Mycobacterium smegmatis*. We explored that MB is an efficient antimicrobial agent against* C. albicans* and* Mycobacterium* with some common (membrane disruption, oxidative stress, and biofilm inhibition) and distinct (cell surface phenotypes, DNA damage, and cell adherence) mechanisms of action.

## 2. Materials and Methods

### 2.1. Media and Growth Conditions

All media chemicals YPD (Yeast Extract Peptone Dextrose) for* C. albicans* and oleic acid/albumin/dextrose/catalase (OADC) for* Mycobacterium,* tetrazolium salt 3-[4, 5-dimethylthiazol-2-yl]-2,5-diphenyltetrazolium bromide (MTT), were purchased from Himedia, Mumbai. Middlebrook 7H9 broth was purchased from BD Biosciences (USA). Calcofluor white (CFW), diethyl pyrocarbonate (DEPC), 4-morpholinepropanesulfonic acid (MOPS), tri-reagent, DNase, and ethidium bromide (EtBr) were obtained from Sigma Chemical Co. (St. Louis, MO, USA). Fresh* C. albicans* (SC5314) was cultured in YPD media (2% dextrose, 2% peptone, and 1% yeast extract) overnight at 30°C before each experiment. Similarly,* Mycobacterium smegmatis*, mc^2^155, was used as a parent wild-type strain for all the experiments. Bacteria were maintained on 7H10 agar supplemented with 10% (v/v) oleic acid/albumin/dextrose/catalase (OADC; BD Difco) and grown in Middlebrook 7H9 (BD Biosciences) broth supplemented with 0.05% Tween-80 (Sigma), 10% albumin/dextrose/catalase (ADC; BD Difco), and 0.2% glycerol (Fischer Scientific) in 100 ml flasks (Schott Duran) and incubated at 37°C till the OD_600_ is equal to 1.0. Stock cultures of log phase cells were maintained in 30% glycerol and stored at −80°C.

### 2.2. Minimum Inhibitory Concentration (MIC)

MIC for* Mycobacterium* was determined by Resazurin microtiter assay (REMA) plate method [10]. Briefly, 100 *μ*L of Middlebrook 7H9 broth was placed at each well of the 96-well microtiter plate following the addition of MB with the remaining media and then subsequently it was serially diluted by 1 : 2. 100 *μ*L of cell suspension (in normal saline to an O.D_600_ 0.1) was added to each well of the plate. Plates were incubated at 37°C for 48 hours. After 48 hours 30 *μ*l of 0.02 per cent resazurin sodium salt solution was added to each well and again incubated for further 2 hours at 37°C.

### 2.3. Static-Cidal Assay

For the static-cidal assay, 3 replicate assay cultures were prepared and* C. albicans* and* M. smegmatis* at 0.1 OD_600_ were seeded to fresh media supplemented with MB at its MIC values and incubated at 30°C and 37°C, respectively. Next day, 100 *μ*l of the same culture was inoculated into the fresh media without MB and incubated for another 24 hrs and OD_600_ was measured with spectrophotometer.

### 2.4. Colony Morphology

For* C. albicans*, colony morphology was determined by plating the cells on YPD agar plates in presence of subinhibitory concentration of MB (25 *μ*g/mL) as determined by growth curve experiments (data not shown) and incubated at 30°C for 2 days. Postincubation, images of the individual colonies were taken at 40x magnification. For* M. smegmatis*, colony morphology was determined as described earlier [11, 12]. Briefly, cells were plated on MB7H10 agar plates supplemented with 10% OADC (BD Difco) and incubated at 37°C for 2 to 4 days in presence of subinhibitory concentration of MB (3.9 *μ*g/mL) as determined by growth curve experiments (data not shown). Postincubation, images of the individual colonies were taken at 10x magnification.

### 2.5. Relative Sedimentation

Cell sedimentation was measured spectrophotometrically as described earlier [13]. Briefly, overnight culture of* C. albicans* was inoculated to 0.1 OD_600_ to both control and MB treated cells and allowed to grow till the OD_600_ reaches 1.0. The OD_600_ from each untreated and treated cultures were measured at each min till 30 minutes. For* M. smegmatis*, cell sedimentation assay was performed as described elsewhere [11, 12]. Cultures at OD_600_ 1.0–1.4 of the control and cells treated with MB at its subinhibitory concentration (3.9 *μ*g/mL) in Middlebrook media supplemented with ADC were adjusted to OD_590_ 1.0 and kept unshaken at 37°C. At 3 and 22 h, the upper 1 ml was removed for OD_590_ measurements. Sedimentation rates were measured by recording the differences in growth from zero time point to 30 min per unit time interval and calculated as described in figure legends.

### 2.6. Propidium Iodide Uptake

Propidium iodide (PI) is membrane impermeable dye and is widely used to differentiate cells that have damaged or nonintact plasma membranes from healthy cells [12, 14]. To evaluate the effect of MB on the plasma membrane of* C. albicans* (approximately 1 × 10^3^ CFUs/ml) and* M. smegmatis*, cells were obtained from exponential phase and exposed to MB (25 *μ*g/ml and 3.9 *μ*g/ml, resp.) for 3 hours at 30°C with gentle shaking. Subsequently, cells were harvested and incubated with 50 *μ*g/ml PI for 15 min in the dark; 10 *μ*l cellular suspension was transferred to a glass slide, covered with coverslip, and examined under fluorescence microscope at 40x (Coslab fluorescence microscope).

### 2.7. ROS Estimation

Cells were stained with DCFDA to detect the presence of reactive oxygen species (ROS) as described elsewhere [15].* C. albicans cells* were grown in YPD overnight in presence of MB at 30°C. After staining with 0.1 *μ*g/mL DCFDA for 15 min, the stained cells were collected and washed thrice with PBS for 5 min. Cells were visualized for presence of ROS by detecting fluorescence (Coslab fluorescence microscope) at magnification 40x. For* M. smegmatis *cells were grown in Middlebrook 7H9 broth in the absence (control) and presence of MB and allowed to grow until it reaches 0.8–0.9 OD. The cells were harvested at 13,000 rpm 15 min and resuspended in 1 ml of media and grown for 1 h at 37°C (Starvation Stress) with shaking. Cells were harvested, washed with PBS 7.4 pH, and resuspended in the same buffer in such a way that each 1 ml of PBS pH 8.5 buffer contains 3 × 10^7^ cells. DCFDA was added in cell suspension with the final concentration of 10 mM and incubated at 37°C for 30 min with continuous shaking. After washing with PBS buffer pH of 7.4 and resuspension in the same buffer, the cells were observed in fluorescence microscope with the excitation and emission value at 488 nm, slit 5 nm and 540 nm, and slit 10 nm, respectively, at 40x. 3 mM H_2_O_2_ was used as positive control and ascorbic acid (AA) at 2.5 mM as antioxidant [12].

### 2.8. DAPI Staining

Cells were stained with DAPI to detect DNA damage as described elsewhere [12, 14]. The cells of exponential phase were inoculated and grown overnight in presence of MB at 30°C for* Candida* and 37°C for* Mycobacterium* and DNA damage was identified by staining with 0.1 *μ*g/mL DAPI for 15 min. The stained cells were collected and washed twice with 1% BSA in PBS for 5 min followed by a five-minute rinse in 0.1% BSA in PBS. Cells were visualized at 100x for* C. albicans *and 40x for* M. smegmatis* with Coslab fluorescence microscope.

### 2.9. Biofilm and Biomass Estimation

*Candida* biofilms were checked on silicon sheet placed in the polystyrene surface of 96-well plates for CFW staining (visualization) and 12-well plates for biomass estimation. An overnight culture was prepared and cell suspension of 1 × 10^7^ cells ml^−1^ was made in PBS and 100 *μ*L was inoculated in each well. The plates were incubated at 37°C for 90 min to adhere the cells on the surface. The wells were gently washed 2-3 times with PBS after 90 min to remove the nonadhered cells. The biofilm was formed by suspending 200 *μ*L of YPD medium along with subinhibitory concentrations of MB (25 *μ*g/ml) and one control without MB to each well of adhered cells to polystyrene 96-well plates and the plates were incubated at 37°C for 24 h. After incubation, wells were washed to remove any planktonic cells and stained with CFW to visualize the formation of biofilms under light microscope. For biomass estimation, the preweighed dried silicon sheets were weighed again after biofilm formation and differences in weights were calculated [13, 16].

*M. smegmatis* biofilm-forming potential was qualitatively and quantitatively analyzed using the microtiter plate method as described elsewhere [11, 12]. Briefly,* M. smegmatis* cultures were grown overnight at 37°C in Middlebrook media, followed by transfer of 100 *μ*l of the media in each well of the 96-well plate with or without the addition of the drug. Cultures that had reached an OD_600_ of 0.1 were diluted (1 : 100) using Middlebrook media and 100 *μ*l of each diluted culture was pipetted into each well of a 96-well flat-bottom microtiter plate and incubated at 37°C for 48 h. The wells were rinsed with water, and 50 *μ*l of 5 mg/ml of MTT was added. Plates were incubated at 37°C for 5 hrs, followed by washing with PBS two times and addition of 100 *μ*l DMSO. The OD_570_ was measured using a spectrophotometer. For qualitative assay,* M. smegmatis* biofilms were formed in Middlebrook 7H9 broth in the absence (control) and presence of MB (3.9 *μ*g/ml) at OD_600_ of 1.0 and incubated for 48 h at 37°C in 12-well plate containing coverslips. The preweight coverslips were rinsed with distilled water and the dry weight was measured after biofilm formation by calculating the difference. 1% CFW was added to each coverslip, and incubated for 10 min at room temperature. Then coverslips were rinsed with PBS and visualized under fluorescent microscope at 40x.

### 2.10. Cell Adherence

For cell adhesion assay on polystyrene plate, the same procedure (as mentioned in biofilm-forming quantitative assay) was followed except that primarily treated and nontreated cells were grown till OD_600_ 1.0 and after washing the nonadhered cells, they were directly quantified with MTT. On the other hand, adherence was also estimated on human oral epithelial cells as described earlier [11–13]. Briefly, cells were grown for 24 h at 37°C and resuspended in 2 ml of sterile PBS (pH 6.8) and washed twice by centrifugation (3000 ×g, 5 min for* C. albicans*, and 10,000 ×g, 15 min for* Mycobacterium*). Author voluntarily donated the epithelial cells via soft scraping of the cheek mucous membrane with sterile cotton swabs, gently stirred, and washed twice with PBS. Adherence assays were developed by mixing 1 ml of each suspension in a test tube and incubated in the presence of MB at 37°C under gentle stirring for 2 h. After incubation, two drops of 0.4% of trypan blue solution and carbol fuchsin (3–5 *μ*l) for* Mycobacterium* only were added to each tube and the mixture was gently shaken. Stained suspensions were examined under light microscopy at 40x and 100x magnifications, respectively.

### 2.11. RNA Isolation and RT-PCR

For RNA isolation [13, 17], the cells were diluted into 50 ml fresh broth at OD_600_ of 0.1 (10^6^ cells ml^−1^) in absence (control) and presence of MB and grown till OD_600_ of 1.0. RNA isolation was performed by Trizol method and reverse transcriptase (RT) PCR was performed as described in the RevertAid H Minus kit (Invitrogen). Briefly, 5 *μ*g isolated RNA was DNase treated at 37°C for 30 min and reaction was terminated by adding 1 *μ*l of 25 mM EDTA and incubated at 65°C for 60 min. RNA was subsequently primed with oligo (dT) 18 for cDNA synthesis at 42°C for 60 min. Reverse transcription reaction was terminated by heating at 70°C for 5 min. The synthesized cDNA product (2 *μ*l) was directly used for PCR amplification reaction (50 *μ*l) using gene specific forward and reverse primers (Table 1). The amplified products were gel electrophoresed and the densities of bands (for genes of interest) were measured and quantified by normalizing to that of the constitutively expressed actin gene (ACT1) in* C. albicans* and 16S gene in* Mycobacterium*.

### 2.12. Statistical Analysis

All experiments were performed in triplicate (*n* = 3). The results were reported as mean ± standard deviation (SD) and analyzed by using Student's *t*-test in which *P* < 0.05 was considered as statistically significant.

## 3. Results and Discussion

### 3.1. MB Is Fungistatic and Mycobacteriostatic in Nature

**Figure 1:**
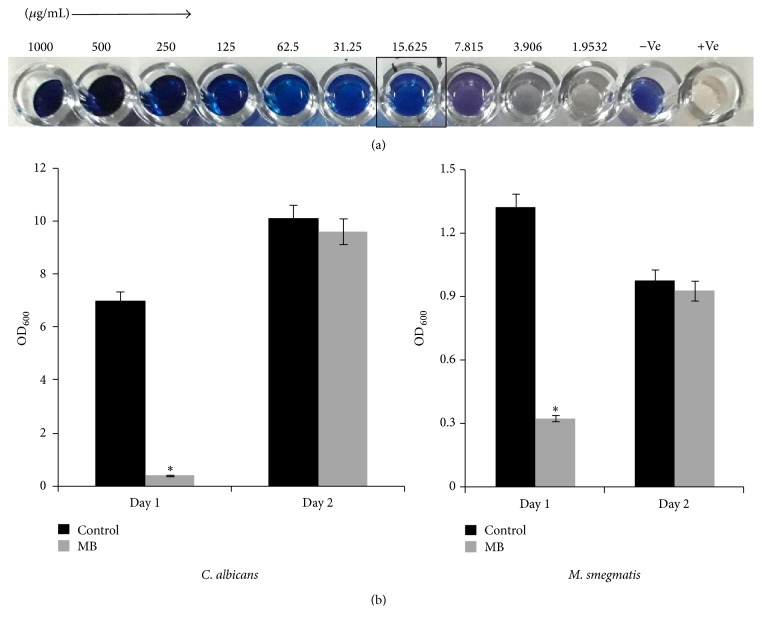
Static/Cidal effect of MB. (a) Drug susceptibility of* M. smegmatis* observed by Resazurin method. (b) Bar graphs depicting the fungistatic (left panel) and mycobacteriostatic (right panel) nature of MB.

In our previous study, we have shown that the* in vitro* antifungal activity of MB is at 100 *μ*g/ml [9]. Here we extended our observation in another frequent human pathogen,* Mycobacterium*. We observed that the antimycobacterial activity of MB at 15.625 *μ*g/ml was sufficient enough to impede the growth of* M. smegmatis* (Figure 1(a)). Interestingly, MB shows its antimycobacterial effect, at much lower MIC value in comparison to its antifungal MIC which was observed at 100 *μ*g/ml. Further, we have checked whether the antimicrobial effect of MB is cidal or static in nature against both the tested organisms. We found that the antimicrobial nature of MB against* Candida* as well as* Mycobacterium* was static in nature (Figure 1(b)).

### 3.2. MB Leads to Impaired Cell Surface Phenotypes in* Mycobacterium* but Not* C. albicans*

**Figure 2:**
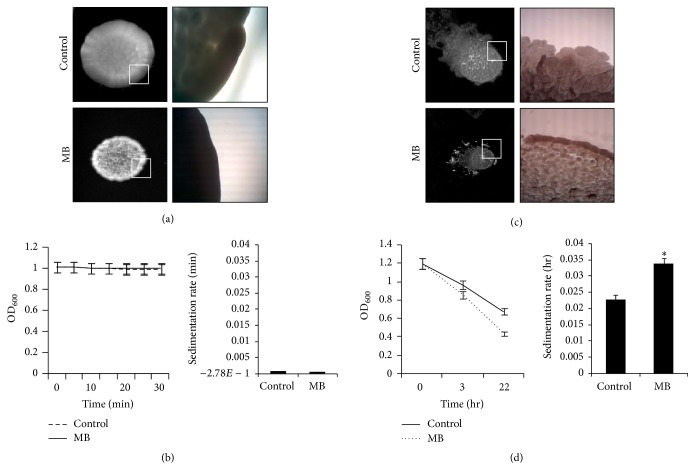
Effect of MB on cell surface phenotypes. (a) Colony morphologies of* C. albicans* on agar plate in absence (control) and presence of MB. (b) Relative sedimentation of* C. albicans* cells. Left panel shows O.D_600_ for untreated (control) and MB treated cells depicted on *y*-axis with respect to time (minutes) on *x*-axis. Right panel shows sedimentation rates expressed as percentage and normalized by considering the untreated control as 100%. Sedimentation rates are depicted on *y*-axis with respect to control and MB on *x*-axis. (c) Colony morphologies of* M. smegmatis* on agar plate in absence (control) and presence of MB. (d) Cell sedimentation of* M. smegmatis*. Left panel shows O.D_590_ of untreated (control) and cell treated with MB cells depicted on *y*-axis with respect to time noted at 3 and 22 hrs on *x*-axis. Right panel shows sedimentation rates per hour on *y*-axis of MB treated cells with respect to control on *x*-axis, calculated by estimating the difference in OD_590_ from 0 till 22 hours per unit time interval and *∗* depicts *P* value < 0.05.

Membrane disruptive effect of MB in* C. albicans* as demonstrated previously [9] prompted us to examine the various cell surface phenotypes more closely. For this, colony morphologies of both untreated and treated (MB)* C. albicans* cells were observed.  Figure 2(a) depicts that there is no appreciable difference in the colony morphology even in the presence of MB except the fact that the colony size was smaller in comparison to control cells. That cell surface phenotypes were not affected in* C. albicans* became further apparent when we used a spectrophotometric based assay to measure sedimentation rates of the cells. Again, the rate of sedimentation of cells grown in the presence of MB showed no considerable difference with respect to the control (Figure 2(b)). Interestingly, colony morphology of* M. smegmatis* appeared as smooth with well-defined borders in the control cells where as in MB treated cells colony appeared as rough and dry with undefined borders (Figure 2(c)). Similarly, the cell sedimentation rate was enhanced in presence of MB for mycobacterial cells (Figure 2(d)). Some studies suggest that this alteration in the cell surface properties can be due the presence of surface antigens, that is, glycopeptidolipids also known as glycolipids [18, 19] in* Mycobacterium* sp., respectively. Thus, it would be interesting to study the glycolipids level in response to MB. Since MTB comprises unique cell envelope components due to the presence of complex lipids that render its drug resistance and pathogenicity [20], altered cell surface phenotypes due to MB are crucial as they may lead to modifications in the cell wall components that could in turn affect cell interactions and various other linked attributes. Further intricate studies are needed to find the reasons for the observed defects in phenotypes.

### 3.3. MB Induced Membrane Perturbation Is Energy Dependent

**Figure 3:**
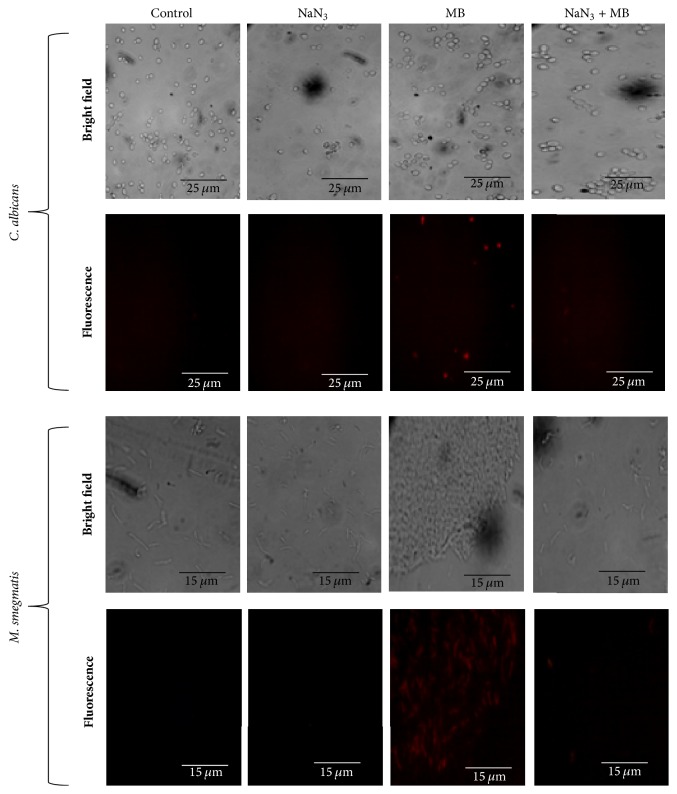
Effect of energy depletion on PI uptake. Fluorescence microscopy of PI for the detection of membrane damage in the presence of MB and sodium azide (ATP inhibitor). Scale bar depicts 25 *μ*m for* C. albicans* and 15 *μ*m for* M. smegmatis,* respectively.

Next, we studied whether the membrane disruptive effect of MB as observed from previous study [14] was energy dependent or not. ATP is known to provide energy in the translocation of the molecule surpassing the membrane. Sodium azide is a known ATP blocker, which diminishes the amount of ATP by targeting the cytochrome-c oxidase and binding in the reduction site of oxygen between the heme a3 iron and CuB region [21–23]. To confirm that the membrane disruptive action of MB is energy dependent, a PI uptake assay was performed with sodium azide treated cells in the presence of MB. PI is a known fluorescent dye which passes through membrane only in conditions like disruption, damage, or any stress and binds to the nucleic acids [14]. We observed that for* C. albicans* and* M. smegmatis*, the cells that were not treated by sodium azide, PI uptake was efficient and showed fluorescence in presence of MB depicting injured membrane. However, in the cells treated with sodium azide no cell was stained with PI (Figure 3). This result not only confirmed our previous hypothesis of membrane disruption in* C. albicans* by MB but also establishes that membrane disruption by MB is energy dependent. Furthermore, this observation also hold true for* Mycobacterium* when similar experiment was performed. Thus, these findings suggest that energy is indispensable for the membrane disruptive effect of MB in both* C. albicans* and* Mycobacterium*.

### 3.4. MB Induces Oxidative Stress

**Figure 4:**
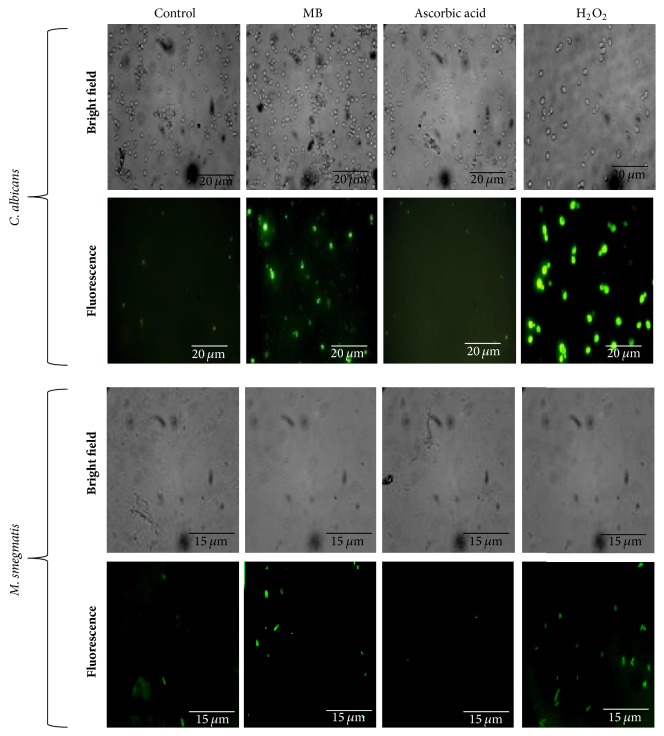
Effect of MB on ROS generation. Fluorescence microscopy of DCFDA for the detection of ROS formation in the presence of MB and reversion after treatment with AA (antioxidant). Scale bar depicts 20 *μ*m for* C. albicans* and 15 *μ*m for* M. smegmatis,* respectively.

Previously, we have suggested that the antifungal action of MB could be due to the altered redox status in* C. albicans* [9]. Moreover, MB has been used in triggering cellular redox metabolism in human derived endothelial cells [24]. Therefore, we extended our observations and confirmed this effect of MB against both* Candida* and* Mycobacterium* by demonstrating the augmentation of the ROS generation. DCFDA is an oxidant sensitive probe which shows green fluorescence upon ROS generation. We explored that MB treated cells showed enhanced level of fluorescence when compared to the untreated cells similar to the cells exposed to hydrogen peroxide. Moreover, the formation of ROS was reverted in presence of antioxidant (AA) to the MB treated cells (Figure 4). During infection, human pathogens must surmount with diverse host-mediated stresses, especially with the antimicrobial properties of macrophages. Macrophages generate antimicrobial reactive oxygen and nitrogen species (ROS and RNS) via NADPH oxidase (NOX2/gp91^phox^) and inducible nitric oxide synthase [25]. Thus, MB leading to oxidative stress in both tested human pathogens could be further exploited for therapeutic purposes.

### 3.5. MB Induces DNA Damage in* Mycobacterium* but Not* C. albicans*

**Figure 5:**
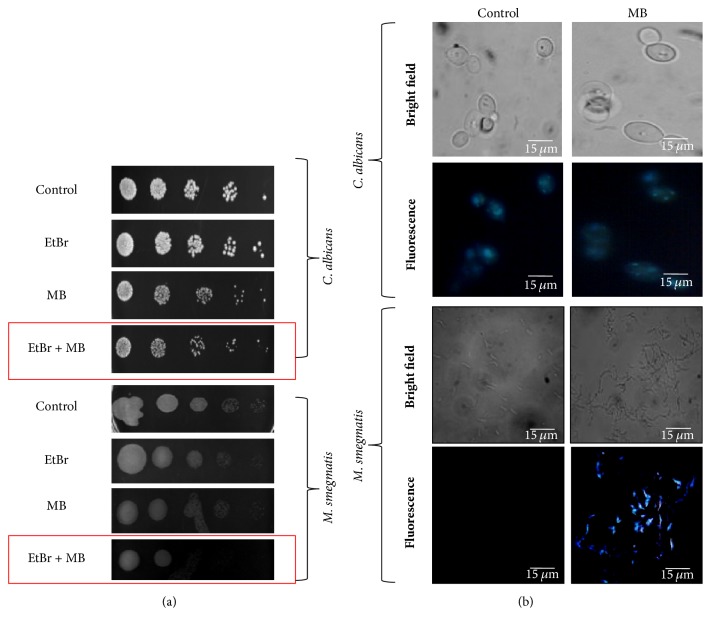
Effect of MB on DNA damage. (a) Phenotypic susceptibility assay in presence of EtBr to assess genotoxicity in* C. albicans* and* M. smegmatis,* respectively. (b) Fluorescence microscopy of DAPI for the detection of DNA damage in the presence of MB. Scale bar depicts 15 *μ*m.

ROS generation leading to oxidative damage is well known for the cause of DNA damage [26]. Moreover, enhanced ROS generation as demonstrated from this study in both the pathogens necessitated exploring the DNA damage in response to MB. To access whether ROS generation leads to the DNA damage in* Candida* and* Mycobacterium*, spot assay was performed using EtBr, a known DNA damaging agent, at a concentration which shows no growth defect in* Candida* cells. We observed that* C. albicans* showed no sensitivity regardless of the presence of MB (Figure 5(a)). However, when similar experiment was performed with* Mycobacterium* cells, we observed sensitivity with MB in the presence of EtBr (Figure 5(a)). To further confirm this preliminary data, DAPI staining was performed which is a fluorescent dye that preferentially binds to the AT site between the minor groove of damaged DNA. Our results demonstrate that in* C. albicans* no difference in the DAPI staining was observed in the MB treated cells when compared to the untreated cells (Figure 5(b)). Interestingly, we found that the* Mycobacterium* cells treated with MB showed blue fluorescence in contrast to no fluorescence in untreated control which could be linked with DNA damage (Figure 5(b)). Despite the fact that the mechanism of DNA damage is not fully elucidated in MTB it is well established that DNA repair mechanisms are necessary for its persistence in the host [27, 28]. These observations confirmed that MB can specifically target the DNA repair response machinery against* Mycobacterium* but not* C. albicans* despite generation of enhanced ROS in both pathogens.

### 3.6. MB Inhibits Biofilm Formation

**Figure 6:**
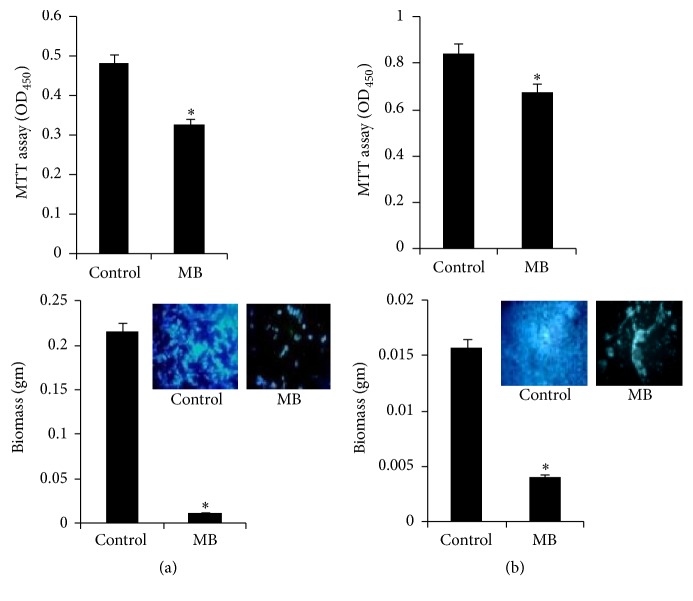
Effect of MB on biofilm formation. (a). Upper panel depicts the effect of MB on biofilm formation of* C. albicans* on polystyrene surface shown as bar graph and quantified by using MTT assay as described in material and methods. OD_450_ nm is depicted on *y*-axis and *∗* depicts *P* value < 0.05. Lower panel depicts the effect of MB on biofilm biomass formed on silicone sheets. Biofilm dry weight is depicted on *y*-axis. Inset represents inhibition in biofilm with fluorescence microscopy images of CFW stained cells in presence of MB. (b). Upper panel depicts the effect of MB on biofilm formation of* M. smegmatis* on polystyrene surface shown as bar graph and quantified by using MTT assay as described in material and methods. OD_450_ nm is depicted on *y*-axis and *∗* depicts *P* value < 0.05. Lower panel depicts the effect of MB on biofilm biomass formed on silicone sheets. Biofilm dry weight is depicted on *y*-axis. Inset represents inhibition in biofilm with fluorescence microscopy images of CFW stained cells in presence of MB.

Biofilms are a defensive forte for microorganisms; within its vicinity they are safe from antibiotic treatment and can create a source of persevering infection. The previously reported data [9] that suggested the inhibition of morphogenetic switching through MB treatment compelled us to study virulence factor, that is, biofilm formation. The biofilm formation was studied by three independent methods, namely, quantitation of biofilm through MTT assay depicting metabolic activity, visualization of CFW stained cells, and dry mass estimation. All the three methods suggest that biofilm formation was significantly suppressed in the presence of MB in both the pathogens (Figures 6(a) and 6(b)). Thus, MB is a potent inhibitor of biofilm formation in both* C. albicans* and* M. smegmatis*.

### 3.7. MB Inhibits Cell Adherence in* Mycobacterium* but Not* C. albicans*

**Figure 7:**
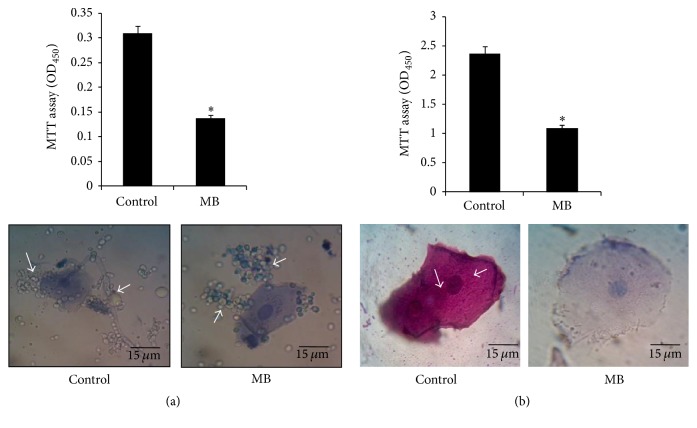
Effect of MB on cell adherence. (a) Upper panel shows the effect of MB on cell adherence on microtiter polystyrene surface of* C. albicans* depicted as bar graph and quantified by using MTT assay. OD_450_ nm is depicted on *y*-axis and *∗* depicts *P* value < 0.05. Lower panel depicts no difference in the epithelial cell adherence in the presence of MB in* C. albicans.* (b) Upper panel shows the effect of MB on cell adherence on microtiter polystyrene surface of* M. smegmatis* depicted as bar graph and quantified by using MTT assay. OD_450_ nm is depicted on *y*-axis and *∗* depicts *P* value < 0.05. Lower panel depicts inhibited epithelial cell adherence in* M. smegmatis* in the presence of MB. Arrows indicate adhered cells which were absent in MB treated cells.

Preliminary to the biofilm formation, the cells have to be attached efficiently to the surface. Thus, microbial cells need to adhere before forming the mature biofilms, thereby making cell adherence another contributing factor in governing virulence [11, 29]. Firstly, we studied the cell adherence of* C. albicans* on microtiter polystyrene surface and human epithelial cells in the presence of MB. Expectedly, the cell adherence was inhibited when observed in polystyrene microtiter surface (Figure 7(a) upper panel). Interestingly, despite having clear effect on the disruption of hyphal formation, we found that MB showed no effect on the adhesive properties of* C. albicans* and the fungal cells could adhere to the human epithelial cells (Figure 7(a) lower panel). When similar experiments were performed for* Mycobacterium*, considerably diminished levels of adherence to both the microtiter polystyrene surface and human epithelial cells were observed when treated with MB (Figure 7(b)). Since alteration in cell property is also linked to biofilm formation and adherence and as a known fact of matter outer portion of nontuberculous mycobacteria (NTM) are attached to the glycolipids [30, 31], hence change in the composition of glycolipids could change the formation of biofilm or adherence of mycobacterium. These observations led us to believe that MB is potent inhibitor of cell adherence only in* Mycobacterium* and not* C. albicans*.

### 3.8. Differential Expression of Genes in Response to MB

**Figure 8:**
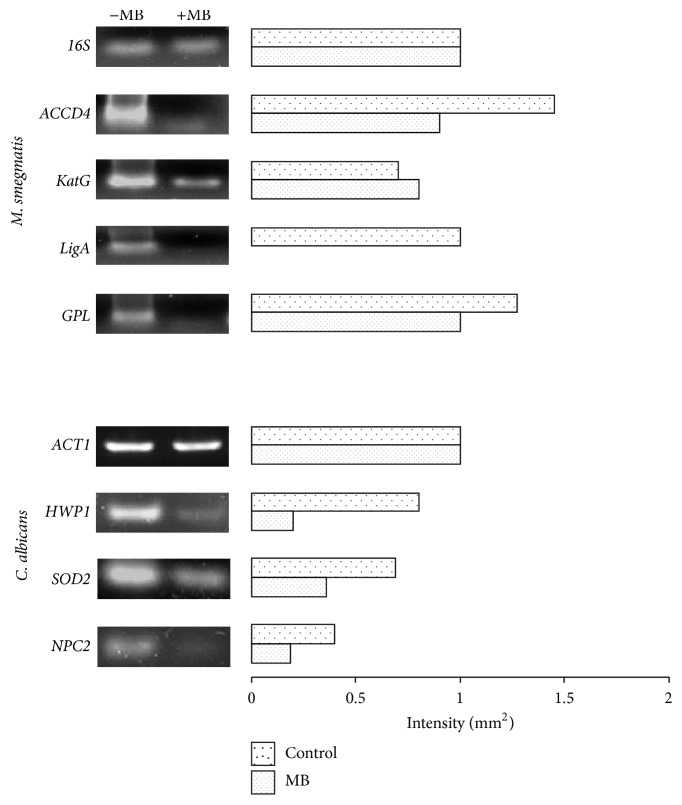
RT-PCR in response to MB. The left panels show transcript levels of lanes (1) Control (−MB), (2) MB. The right panel shows the quantitation (density expressed as intensity/mm^2^) of the respective transcripts normalized with constitutively expressed 16S and* ACT1* genes for* Mycobacterium* and* C. albicans,* respectively.

The disrupted phenotypes reported in this study were validated by RT-PCR. Mycolic acid is a major constituent of the mycomembrane and hallmark of mycobacteria having crucial role in the permeability of the membrane [32]. In our study, we found downregulated* ACCD4* gene in response to MB (Figure 8) which is essential for the synthesis of mycolic acid and survival of* M. smegmatis* [33]. Similarly, in* Mycobacterium*, catalase-peroxidase (KatG) is an important protein responsible for activation of the isoniazid. It is already known that the level of KatGp is elevated in oxidative stress [34]. Concomitant with this, we found an upregulation of* KatG* gene in* Mycobacterium* when treated with MB (Figure 8). Similarly, we found the downregulation in the* LigA* gene (Figure 8) which is known for its activity in DNA repair and its replication [35].* GPL* is a gene responsible for the formation of biofilm, colony morphology, and sliding motility [36, 37]. In our study, we found that* GPL* is downregulated in presence of MB (Figure 8) which was consistent with MB inhibiting the formation of biofilm and colony morphology alteration.

In* Candida*, hyphal wall protein 1 (Hwp1p) is an important factor to show virulence expression like morphogenetic switching and biofilm formation [38]. We have found that* HWP1* gene was downregulated in* Candida* cells when treated with MB (Figure 8). Superoxide dismutase* (SOD2)* gene is an important gene responsible for the protection against oxidative stress [39]. Through RT-PCR, it was confirmed that* SOD2* gene was downregulated in* C. albicans* when treated with MB (Figure 8). Niemann-Pick C2* (NPC2)* gene is involved in sterol transportations through protein membrane interaction [40]. It was found that* NPC2* gene was downregulated when treated with MB (Figure 8) which was consistent with the MB induced membrane disruption. Thus, all the RT-PCR results showed good correlation with the MB induced disrupted phenotypes in* Mycobacterium* as well as* C. albicans*.

## 4. Conclusion

**Figure 9:**
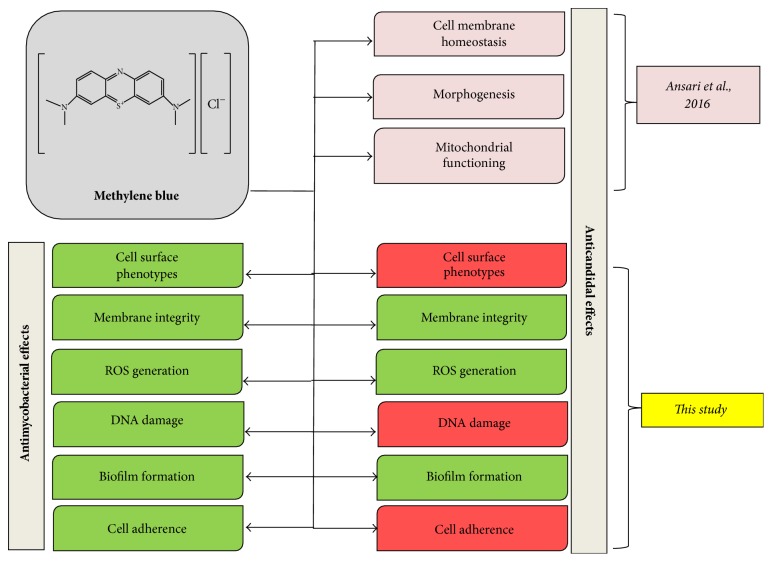
Summary of altered mechanisms in* C. albicans* and* M. smegmatis* cells after MB treatment. Green and red color depict affected and unaffected phenotypes, respectively.

Given the limited number of available antifungal and antimycobacterial therapies with concomitant increase in drug resistance, the demand for the search of new, safe, and effective antifungal and anti-TB treatments are inevitable. Considering the fact that MB is FDA approved dye that is already used for various therapeutic options, the distinct antifungal and antimycobacterial mechanisms of MB (Figure 9) presented in this study will aid in comprehending further studies on MB to be exploited as promising drug for future.

## Figures and Tables

**Table 1:** List of primers used in the study for *M. smegmatis* and *C. albicans,* respectively.

S. number	Gene	Sequence
1	16S	5′-GGCGAACGGGTGAGTAACA-3′
		3′-GCCCTGCACTTTGGGATAAG-5′
2	KATG	5′-GCCACCCAGGAAGAGACC-3′
		3′-GCAGGTTGACGAAGAAGTCC-5′
3	LIGA	5′-AACACCAGTTCCGGTACTACGT-3′
		3′-CGAGCGCCTGCAGTT-5′
4	ACCD4	5′-GCACTCGGAATGCCCTTCTTCTC-3′
		3′-ACGAACAAGACCACCGCTGAACTC-5′
5	GPL	5′-CATGATCCCCGAGGAGCAC-3′
		3′-TTGCCGTTCAAGTACCTCGG-5′
6	ACT1	5′-TTTTGACCTTGAGATACCCA-3′
		3′-GGAGCTCTGAATCTTTCGTT-5′
7	HWP1	5′-ACTACCCACAACAACCACAA-3′
		3′-GCAGATGATGATTCTGAAGTG-5′
8	SOD	5′-TCAGATCATCATCTCGTGTTT-3′
		3′-TCTTCTTTCAGCTTCCTTCC-5′
9	NPC2	5′-GAACTTGGCAATTGTTACCC-3′
		3′-CAGGGAATATAATTGTAGCAG-5′
